# Combination of anti-angiogenic therapy and immune checkpoint blockade normalizes vascular-immune crosstalk to potentiate cancer immunity

**DOI:** 10.1038/s12276-020-00500-y

**Published:** 2020-09-11

**Authors:** Won Suk Lee, Hannah Yang, Hong Jae Chon, Chan Kim

**Affiliations:** 1 Laboratory of Translational Immuno-Oncology, Seongnam, Korea; 2 Medical Oncology, CHA Bundang Medical Center, CHA University School of Medicine, Seongnam, Korea

**Keywords:** Cancer immunotherapy, Cancer microenvironment, Tumour angiogenesis, Tumour immunology, Targeted therapies

## Abstract

Cancer immunotherapy with immune checkpoint inhibitors (ICIs) has revolutionized the treatment of advanced cancers. However, the tumor microenvironment (TME) functions as a formidable barrier that severely impairs the efficacy of ICIs. While the crosstalk between tumor vessels and immune cells determines the nature of anti-tumor immunity, it is skewed toward a destructive cycle in growing tumors. First, the disorganized tumor vessels hinder CD8^+^ T cell trafficking into the TME, disable effector functions, and even kill T cells. Moreover, VEGF, the key driver of angiogenesis, interferes with the maturation of dendritic cells, thereby suppressing T cell priming, and VEGF also induces TOX-mediated exhaustion of CD8^+^ T cells. Meanwhile, a variety of innate and adaptive immune cells contribute to the malformation of tumor vessels. Protumoral M2-like macrophages as well as T_H_2 and Treg cells secrete pro-angiogenic factors that accelerate uncontrolled angiogenesis and promote vascular immaturity. While CD8^+^ T and CD4^+^ T_H_1 cells suppress angiogenesis and induce vascular maturation by secreting IFN-γ, they are unable to infiltrate the TME due to malformed tumor vessels. These findings led to preclinical studies that demonstrated that simultaneous targeting of tumor vessels and immunity is a viable strategy to normalize aberrant vascular-immune crosstalk and potentiate cancer immunotherapy. Furthermore, this combination strategy has been evidently demonstrated through recent pivotal clinical trials, granted approval from FDA, and is now being used in patients with kidney, liver, lung, or uterine cancer. Overall, combining anti-angiogenic therapy and ICI is a valid therapeutic strategy that can enhance cancer immunity and will further expand the landscape of cancer treatment.

## Cancer: Combination treatment targeting tumor blood vessels and immunity

Combining anti-angiogenesis drugs that reduce the growth of blood vessels and immune checkpoint inhibitors that promote the activation of cancer-killing immune cells offers a promising new therapeutic regimen for patients with cancer. In a review article, a team led by Chan Kim and Hong Jae Chon from the CHA University School of Medicine in Seongnam, South Korea, discuss the molecular crosstalk between blood vessels and immune cells in the tumor microenvironment, a biological interconnectedness that provides a compelling rationale for the dual treatment strategy. The researchers summarize preclinical and clinical data demonstrating the potential of combining immunotherapy with treatment targeting blood vessel growth across a range of tumor types. These data have so far led to regulatory approvals for patients with cancers of the lung, kidney, liver and endometrium.

## Introduction

For the last decade, immune checkpoint inhibitors (ICIs) have revolutionized the treatment landscape of advanced cancers. ICIs rejuvenate dysfunctional or exhausted cytotoxic T cells (CTLs) to exert potent anti-tumor effector functions, thereby enabling effective and durable control of previously refractory cancers^1–3^. However, despite these advances, only 20–30% of patients with cancer respond to ICI treatments, and it is difficult to predict these responders before treatment because the immune system is too complex to interpret with a single biomarker. Among the various determinants of cancer immunity, the tumor microenvironment (TME) functions as a major obstacle that severely impairs the efficacy of ICIs^2,4^. Within the TME, the interplay between tumor vessels and protumoral immune cells generates a vicious cycle that severely disturbs anti-cancer immunity and promotes tumor progression; abnormal tumor neovessels foster protumoral immune cell evasion, which in turn bolsters tumor angiogenesis^2,5–7^. This aberrant immune-vascular crosstalk not only generates an endothelial barrier that hinders T-cell infiltration into the tumor but also impairs T-cell effector functions and even leads to T cell apoptosis within the TME^2,4,8–10^. Thus, targeting the tumor vasculature can be a potential solution to enhance anti-cancer immunity and overcome resistance to ICIs.

Here, we summarize the emerging evidence of the mutual regulation of blood vessels and immune cells within the TME and provide a rationale for a combination immunotherapy that targets both tumor vessels and immunity. In addition, we highlight the recent major clinical breakthroughs in cancer immunotherapy that have proven the validity of combining anti-angiogenic therapy and ICIs.

## Tumor vasculature negatively impacts cancer immunity at multiple steps

**Fig. 1:**
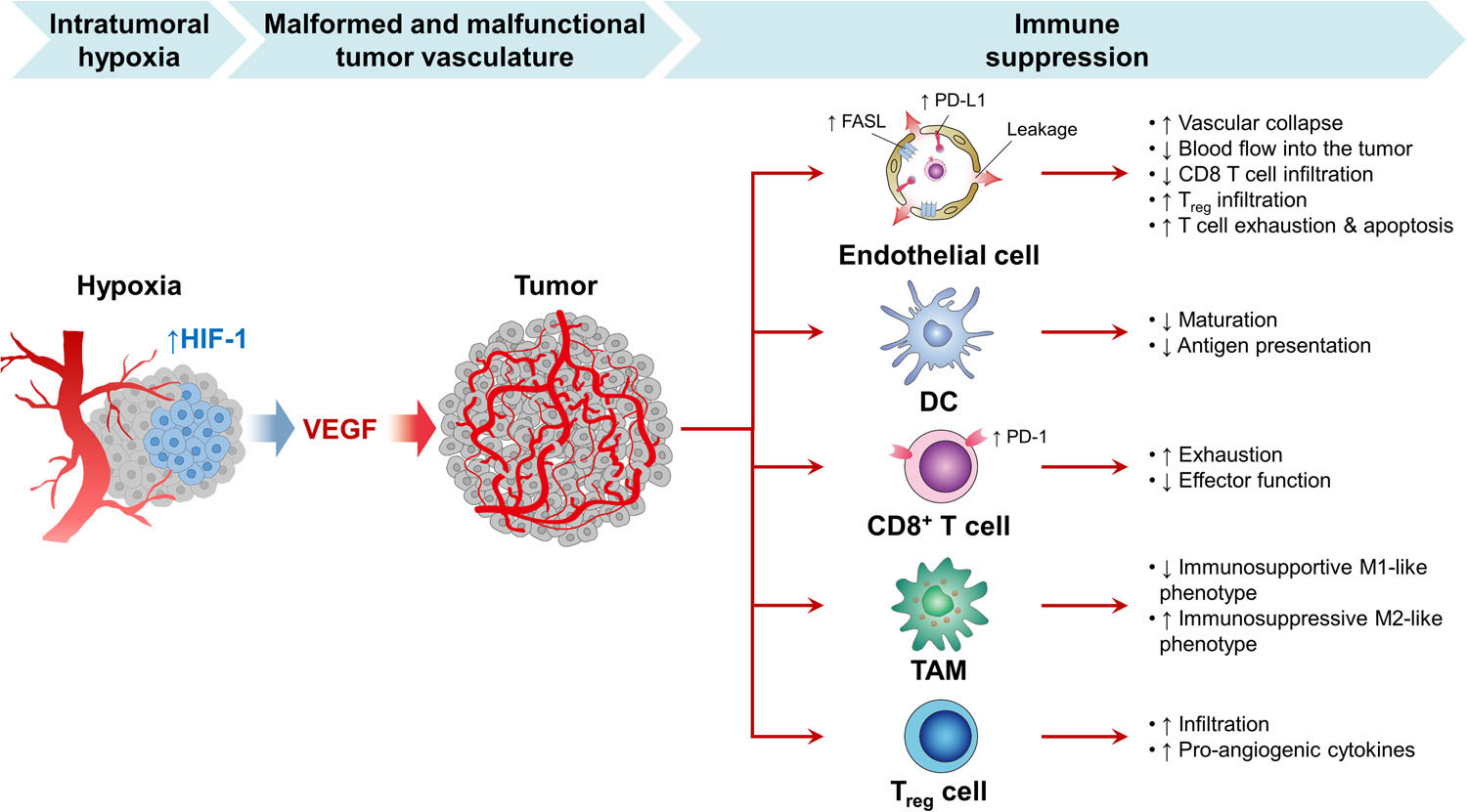
The abnormal tumor vasculature elicits immune suppression in the tumor microenvironment. Tumor cells rapidly outgrow their blood supply, leading to hypoxia and acidosis in the tumor microenvironment (TME), which in turn promotes immunosuppressive mechanisms. Hypoxia stimulates HIF-1 and thereby upregulates VEGF. VEGF induces tumor angiogenesis, resulting in the malformed and malfunctional vasculature. Tumor endothelial cells exhibit immunosuppressive characteristics, such as PD-L1 expression, which enhance the exhaustion and apoptosis of T cells. Dendritic cell (DC) maturation is suppressed, resulting in interruption of T cell priming by impaired antigen presentation. In addition, TOX-mediated transcriptional reprogramming severely exhausts CD8^+^ T cells. Furthermore, tumor-associated macrophages (TAMs) polarize from an immunosupportive M1-like phenotype to an immunosuppressive M2-like phenotype. Regulatory T (T_reg_) cells also accumulate within the TME to promote tumor angiogenesis.

Tumor growth depends on adequate oxygen and nutrient supply from the blood vessels^11,12^. However, in rapidly progressing tumors, tumor growth often exceeds the supply from the existing vasculature, resulting in intratumoral hypoxia. Hypoxia activates the angiogenic master switch, called hypoxia-inducible factor-1 (HIF-1), and upregulates vascular endothelial growth factor (VEGF) in tumors^13–15^. VEGF, in turn, promotes tumor angiogenesis by inducing the proliferation and survival of endothelial cells (ECs), forming a myriad of malformed and malfunctional neovessels within the tumor^4,13,16^. These tumor vessels disturb the establishment of active anti-cancer immunity at multiple steps and restrain the efficacy of ICI treatment against the tumor (Fig. 1)^2–4,17,18^.

First, an abnormal tumor vasculature serves as a physical barrier for CTLs. These tumor vessels are a chaotic network of immature microvessels without structural hierarchy, resulting in inefficient blood distribution within the tumor^12,19,20^. They are very leaky, have loose interconnections among the endothelium, and lack adequate wrapping by pericytes and basement membrane. Therefore, a large volume of fluid leaks from these hyperpermeable tumor vessels and accumulates in the TME, generating high interstitial fluid pressure that collapses tumor blood vessels and severely hinders blood flow into the tumor. Therefore, most tumor vessels fail to deliver enough oxygen, nutrients, and effector cells deep into the tumor. Above all, tumor-specific CTLs in the bloodstream cannot infiltrate into the TME due to this abnormal tumor vasculature and, as a result, are not able to eradicate tumor cells.

Second, the tumor vasculature disables and kills CTLs by expressing various immunosuppressive molecules, such as PD-L1 and Fas ligand (FasL, also known as CD95L). PD-L1 on the tumor endothelium can be upregulated by chronic hypoxia or interferon-γ (IFN-γ) and inactivate T cells within the tumor vascular lumen, which become functionally anergic before migrating across the vessel wall and entering the TME^21^. Moreover, a substantial proportion of tumor vessels overexpress FasL, a death ligand for activated T cells, on their surface. FasL on tumor vessels selectively kills CTLs but not regulatory T cells (Tregs) because of their high expression of c-FLIP, resulting in rare CTL but predominant Treg infiltration in the TME^21^.

VEGF, the critical driver of tumor angiogenesis, is a potent immunosuppressive factor in both innate and adaptive anti-tumor immunity. VEGF in the TME interferes with the maturation of dendritic cells (DCs) from immature precursors, thereby interrupting T-cell priming against tumors^22^. VEGF binding to VEGFR1 hinders the maturation of DCs through the inactivation of NF-kB signaling in murine tumor models^23^. Moreover, increased plasma VEGF levels correlate with an increased number of immature DC precursors but a decreased number of DCs in the peripheral blood of patients with cancer. Anti-VEGF treatment reverses this VEGF-mediated immunosuppression on DCs; it not only decreases immature progenitors but also increases mature DCs. VEGF also plays an immunosuppressive role in the TME by accumulating Tregs and repolarizing tumor-associated macrophages (TAMs) to M2-like phenotypes^2,5^. In addition, VEGF induces the TOX-mediated exhaustion program in CD8^+^ CTLs^24^. TOX is a recently elucidated transcription factor for T-cell development that plays an important role in T-cell priming^25^. In the TME, excess VEGF upregulates TOX expression in CD8^+^ T cells and initiates TOX-mediated transcriptional reprogramming toward the exhausted state and upregulates multiple checkpoint inhibitor receptors on these T cells^24^. Notably, conditional knockout of VEGFR2 in CD8^+^ T cells could downregulate TOX and reactivate tumor-specific CD8^+^ T cells, indicating the potential of VEGF/VEGFR2 axis-targeted therapy in rejuvenating exhausted T cells^24^.

In addition to VEGF, other pro-angiogenic factors are also involved in tumor angiogenesis and immune suppression within the TME. Angiopoietin (ANGPT) binding to the receptor tyrosine kinase Tie-2 regulates tumor angiogenesis and vascular integrity. While ANGPT1 stabilizes the tumor vasculature through recruitment of pericytes to growing vessels, ANGPT2 strongly promotes excessive angiogenic sprouting with reduced pericyte coverage. Furthermore, ANGPT2 negatively influences tumor immunity by recruiting M2-like TAMs and Tie-2-expressing monocytes/macrophages (TEMs) into tumors; TEMs then promote Treg infiltration via IL-10 but suppress CTL activation^3,26^. Transforming growth factor-β (TGF-β) is another important factor that regulates the proliferation of pericytes and ECs and induces different angiogenic responses depending on the balance between ALK1 and ALK5 signaling. Notably, TGF-β/ALK1 signaling activates Smad1/5, which promotes EC proliferation, migration, and tube formation. Moreover, TGF-β inactivates tumor immunosurveillance by inhibiting NK and T cells, leading to tumor progression^27,28^. Placental growth factor (PlGF), another member of the VEGF family, is an important regulator of the pro-angiogenic phenotype within the TME. PlGF directly interacts with VEGFR1 to stimulate tumor angiogenesis, increase vascular permeability, and promote TAM repolarization to the M2 phenotype. PlGF blockade induces vessel normalization and macrophage polarization from the M2-like to M1-like phenotype^29,30^.

Last, tumor blood vessels foster immune evasion by preferentially recruiting immunosuppressive immune cells into the TME. As noted above, abnormal tumor vessels give rise to a hypoxic TME. This promotes the secretion of soluble chemotactic factors, such as CCL2, CCL22, CCL28, CXCL8, and CXCL12, which facilitate the recruitment of immunosuppressive cells, such as myeloid-derived suppressor cells (MDSCs), M2-like TAMs, and Tregs, into the tumor^2,5^. These immune cells collaborate with tumor cells to suppress the magnitude of the anti-tumor immune response in the TME, thereby promoting tumor progression.

Several clinical studies also support a possible resistance mechanism against immunotherapy by VEGF. The liver is one of the most well-known hypervascular organs and has a high VEGF level compared with other organs^31^, and cancer patients with liver metastasis showed poorer clinical benefit from anti-PD-1 monotherapy in non-small cell lung cancer (NSCLC), melanoma, or kidney cancer^32^. Moreover, a recent study revealed that liver metastatic lesions have the lowest response rate compared with other organs in organ-level response analyses of patients with NSCLC treated with anti-PD1 therapy^33^. Intriguingly, patients with liver metastasis showed significantly less CD8^+^ T cell infiltration into the tumor compared with those without liver metastasis^32^, suggesting VEGF-mediated T cell exclusion within tumors and explaining the attenuated response to anti-PD-1 therapy in VEGF-high tumors. Such results warrant further investigation of the immunoregulatory effects of VEGF blockade on VEGF-high tumor lesions such as liver metastases.

## Targeting the tumor vasculature: from vascular destruction to vascular normalization

The original concept of anti-angiogenic therapy simply focused on inhibition of new vessel formation and the destruction of established vessels to starve tumor cells to death. However, this concept leads to a therapeutic paradox in which excessive anti-angiogenic therapy could cut not only the intratumoral blood supply but also the delivery of concurrent anti-cancer drugs and anti-cancer immune cells, such as CTLs into the tumor. Moreover, additional concerns were raised from preclinical studies suggesting that complete blockade of intratumoral blood flow could result in extreme hypoxia within the TME, which could accelerate local invasion and distant metastases of tumor cells and even induce severe immunosuppression within the TME^34^. To resolve this paradox, Jain et al. proposed a vascular normalization theory in which a judicious intensity of anti-angiogenic therapy could result in an equilibrium between anti-angiogenic and pro-angiogenic signals within the TME—vascular normalization status—and enable efficient delivery of oxygen, drugs, and anti-cancer immune cells into the tumor, rather than inducing excessive destruction of vessels and intratumoral hypoxia^35,36^. In other words, stronger therapy may not always be better; lower, optimal-dose anti-angiogenic treatment could be more advantageous than higher-dose therapy to establish a favorable TME leading to the therapeutic benefit.

Consistently, Huang et al. reported that adequate intensity anti-angiogenic therapy is critical to alleviate intratumoral hypoxia and establish an immunosupportive TME^37^. In their study, high-dose anti-VEGFR2 treatment aggravated intratumoral hypoxia and restrained the infiltration of CD8^+^ T cells into the TME, thereby suppressing anti-cancer immunity. On the other hand, low-dose anti-VEGFR2 treatment normalized tumor vessels with increased pericyte coverage, improved tumor perfusion, and eventually promoted the infiltration of CD8^+^ and CD4^+^ T cells into the tumor. Furthermore, Jung et al. also revealed that higher-dose anti-VEGFR2 therapy could result in intratumoral immunosuppression mediated by Ly6C^low^ monocytes and Ly6G^+^ neutrophils while impairing the adaptive immune cells within the TME^38,39^. In line with these results, Rivera et al. also reported that higher-dose anti-angiogenic therapy could activate PI3K signaling in myeloid cells that promotes immune suppression and neovascularization^40^.

While evidence supporting the vascular normalization theory has been accumulating over the past decade, questions still remain. First, it is not clear whether this hypothesis could be universally applied using all anti-angiogenic agents to all stages of carcinogenesis. Next, the therapeutic dose of anti-angiogenic therapy in clinical practice varies depending on the type of tumor and its clinical setting. For example, a lower dose (5 mg/kg) of bevacizumab (anti-VEGF-A) is used in colorectal cancer, while a higher dose (15 mg/kg) is used in lung cancer. However, it is not clear which dose corresponds to either the vascular normalizing dose or vascular destructing dose in patients with cancer. Therefore, further preclinical and clinical studies are warranted to optimize anti-angiogenic therapy in the era of cancer immunotherapy to open the vascular normalization window within the TME and enhance anti-tumor immunity.

## Immune cells play versatile roles in the regulation of tumor angiogenesis

**Fig. 2:**
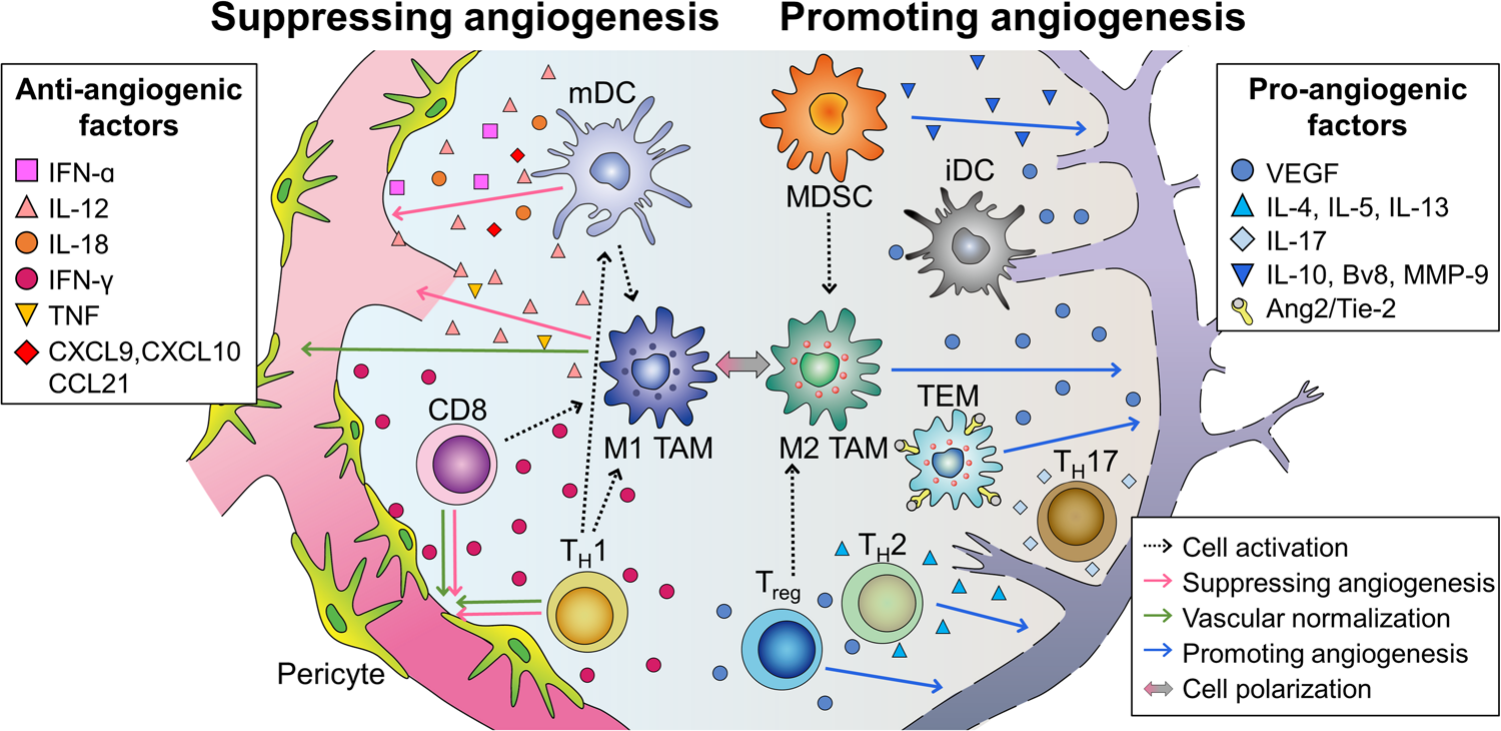
A variety of immune cells orchestrate tumor angiogenesis. Immune cells directly influence the phenotypes and functions of tumor vessels through various cytokines. Innate immune cells, such as mature dendritic cells (mDCs) and M1-like TAMs, produce cytokines (IFN-α, IL-12, IL-18, or TNF) and chemokines (CXCL9, CXCL10, or CCL21) that suppress tumor angiogenesis. Meanwhile, adaptive immune cells, such as CD8^+^ T cells and T helper 1 (T_H_1) cells, secrete IFN-γ, a potent cytokine that inhibits angiogenesis and induces vascular normalization in the TME. However, immature DCs (iDCs), myeloid-derived suppressor cells (MDSCs), M2 TAMs and Tie2-expressing macrophages (TEM) significantly promote tumor angiogenesis by secreting VEGF, IL-10, Bv8, and MMP-9. Moreover, T_reg_, T_H_2, and T_H_17 cells can also release pro-angiogenic factors such as VEGF, IL-4, IL-5, IL-13, and IL-17. In addition to direct effects on tumor vasculature, immune cells regulate tumor vasculature indirectly by communicating and polarizing with each other. mDC, CD8, and T_H_1 cells can skew macrophage polarization away from the M2 to the M1 phenotype. However, MDSCs and T_reg_ cells can reprogram TAMs from M1 to M2.

Immune cells orchestrate the whole process of tumor angiogenesis via both direct and indirect mechanisms (Fig. 2). Numerous pro- or anti-angiogenic factors derived from immune cells directly influence tumor vessels and determine the endothelial phenotype and function^5,7,41,42^. Moreover, certain types of immune cells can communicate and polarize other types of immune cells to be either pro-angiogenic or anti-angiogenic, indirectly affecting the quantity and quality of tumor angiogenesis^41,43^.

Macrophages exhibit notable plasticity in the regulation of tumor angiogenesis. They constitute functionally heterogeneous innate immune cells depending on the type of secreted cytokines and growth factors. Notably, they modify their transcriptional program in response to stimuli from the TME along a continuous spectrum, with an M1- and M2-like phenotype at both extremes; M1-like TAMs suppress tumor angiogenesis, whereas M2-like TAMs promote tumor angiogenesis^41,42,44–46^.

M1-like TAMs suppress sprouting angiogenesis and induce vessel maturation by secreting anti-angiogenic cytokines, such as interleukin (IL)-12 and TNF-α^47,48^. Intriguingly, M1-like TAM-derived IL-12 polarizes macrophages toward the M1 phenotype, thereby generating a positive feedback loop for the anti-angiogenic M1 phenotype. Accordingly, immunotherapy with IL-12 not only reduces microvessel density but also enhances M1 macrophage polarization in tumors^48–50^.

M2-like TAMs promote tumor angiogenesis by producing pro-angiogenic growth factors (VEGF, EGF, FGF family, and PDGF-b), angiogenic CXC chemokines (CXCL8/IL-8 and CXCL12, also known as SDF-1), and angiogenesis-associated factors (TGF-b, TNF-α, and thymidine phosphorylase)^51,52^. These factors not only enhance the migration and proliferation of ECs but also further skew macrophage polarization away from M1 to the tumor-promoting M2 phenotype^44,47,48^. As M2-like TAMs are a more dominant population than M1-like TAMs in most advanced tumors, pharmacological depletion of macrophages with clodronate- liposome generally suppresses tumor angiogenesis and tumor growth in transplanted tumor models^53,54^.

Another distinct subtype of macrophages that was defined relatively recently is TEMs, which also plays an important role in encouraging tumor angiogenesis^55–57^. When Tie-2 on the surface of TEMs binds to angiopoietin-2 secreted from endothelial and tumor cells, a strong angiogenic switch is turned on in the TME. Consistently, tumors fail to sustain angiogenesis in the absence of Tie-2 signaling in macrophages^58^. In addition, selective depletion of Tie-2 expression in macrophages induces tumor vascular normalization and the regression of established tumors, supporting the critical role of TEMs during tumor angiogenesis^56,57^.

DCs, another important innate immune component of the TME, can regulate tumor angiogenesis depending on their maturation status^59^. Mature DCs can be classified into two major subtypes, conventional DCs (cDCs) or plasmacytoid DCs (pDCs)^60,61^. Mature cDCs suppress tumor angiogenesis by secreting anti-angiogenic cytokines, namely, IL-12 and IL-18, and angiostatic chemokines, including CXCL9, CXCL10, and CCL21^62–64^. In contrast, mature pDCs secrete interferon-α (IFN-α), which inhibits the proliferation and motility of ECs and increases anti-angiogenic cytokines and chemokines in the tumor^65,66^. Unfortunately, in the TME, the most frequent subset of DCs is immature DCs (iDCs) because cancer cells can preferentially recruit iDCs from peripheral blood vessels by releasing a number of cytokines (e.g., VEGF, β-defensin, CXCL12, HGF, and CXCL8)^64,67–69^.

MDSCs, a heterogeneous population of immature myeloid cells, can augment tumor angiogenesis via several mechanisms. MDSCs enhance angiogenesis by increasing IL-10 and decreasing IL-12 in the TME^43,45,46,70^. Furthermore, MDSCs can promote angiogenesis by producing Bv8 and MMP-9. MDSC-derived Bv8 can directly promote neovessel formation via endocrine gland-derived VEGF1 (EG-VEGF1) and VEGF2 (EG-VEGF2) and can further accumulate MDSCs within the tumor^71–73^. Therefore, neutralizing antibodies against Bv8 significantly reduce tumor vascular density and the number of tumor-infiltrating MDSCs^72^. Simultaneously, MMP-9 can induce tumor angiogenesis by releasing biologically active VEGF from the extracellular matrix of the TME. Accordingly, MMP-9-deficient MDSCs fail to induce tumor angiogenesis^46,73^. Third, unlike other immune cells, some MDSCs can differentiate into EC-like cells. These EC-like MDSCs express endothelial markers, such as CD31 and VEGFR2, and have the ability to integrate into the tumor vasculature^45,46,73^.

Adaptive immune cells are also critical players in the orchestration of tumor angiogenesis by directly affecting EC biology and indirectly modulating myeloid cell phenotypes. Among adaptive immune cells, CD8^+^ CTLs play a critical role in suppressing tumor angiogenesis by secreting IFN-γ^74,75^. IFN-γ directly inhibits the proliferation and migration of human endothelial cells and secretes IFN-inducible protein 10 (IP-10) and monokine induced by IFN-γ (MIG). These cytokines also react with CXCR3, restraining the proliferation of endothelial cells and tumor vascularization^74,76^. Furthermore, IFN-γ signaling downregulates VEGF-A but upregulates CXCL9, CXCL10, and CXCL11, which collectively stimulate vascular maturation by enhancing pericyte recruitment along ECs^74,77,78^. Another important aspect of IFN-γ in tumor angiogenesis is the reprogramming of TAMs from M2- to M1-like TAMs. Hyperactive IFN-γ/STAT1 signaling promotes M1-like TAM reprogramming, leading to vascular remodeling and consequent tumor eradication^7,77,79^.

In addition to CD8^+^ CTLs, CD4^+^ T helper 1 (T_H_1) cells assist in tumor vessel normalization by producing IFN-γ in the TME. Depletion of CD4^+^ T_H_1 cells decreases pericyte coverage and increases malformed tumor vessels in multiple mouse tumor models, whereas activation of CD4^+^ T cells improves vessel normalization^7,80,81^. T_H_1 cells also polarize M2-like TAMs to M1-like TAMs and induce DC maturation in the TME, which suppresses tumor angiogenesis^82,83^.

In contrast to CD8^+^ CTLs and T_H_1 cells, T_H_2 cells promote robust tumor angiogenesis. T_H_2 cells expressing IL-4, IL-5, and IL-13 recruit M2-like TAMs through STAT-6 activation and promote tumor angiogenesis^41,50,77,84^. T_H_17 cells, another subtype of CD4^+^ T cells, are associated with increased angiogenesis in various human cancers. The expression of IL-17 by T_H_17 correlates with the infiltration of ECs and abnormal tumor vasculature^41,77,85,86^.

Tumor-infiltrating Treg cells also play a critical role by sustaining angiogenesis directly through VEGF secretion and supporting endothelial cell recruitment and expansion^83,87^. Furthermore, Tregs promote angiogenesis indirectly by restraining the activity of T_H_1 cells and by triggering the activation of M2-like macrophages^42^. In ovarian cancer, hypoxia results in CCL28 upregulation, leading to a robust increase in Treg infiltration, VEGF and blood vessels, whereas depletion of Tregs reduces intratumoral VEGF levels and tumor angiogenesis^18,81^.

## Preclinical studies provide a rationale for combining anti-angiogenesis therapy with ICIs

The interactions between tumor immunity and angiogenesis suggest that tumor vascular remodeling could enhance the efficacy of cancer immunotherapy. Emerging preclinical evidence demonstrates the potential of combining immunotherapy with vascular-targeting treatment^24,37,75,88–91^. Allen et al. demonstrated that anti-angiogenic therapy with anti-VEGFR2 enhances the efficacy of anti-PD-L1 immunotherapy in pancreatic neuroendocrine tumor (RT2-PNET), mammary carcinoma (MMTV-PyMT), and glioblastoma (NFpp10-GBM) models^88^. Anti-VEGFR2 treatment upregulated the expression of PD-L1 via IFN-γ secretion by CD8^+^ T cells to potentially enhance the sensitivity of anti-PD-L1 therapy in tumors. Furthermore, the combination of anti-angiogenic and immunotherapy increased pericyte coverage and normalized tumor vessels, promoting intratumoral infiltration of activated T cells. In addition to vascular normalization, the vessel phenotype represents the characteristics of high endothelial venules (HEVs), which are morphologically thickened with plump endothelial cells (ECs) and functionally more specialized in lymphocyte extravasation than other tumor ECs. Notably, the LTβR signaling pathway is involved in the generation of intratumoral HEVs after combined treatment with anti-VEGFR2 and anti-PD-L1. Therefore, these results suggest that anti-angiogenic therapy could improve the efficacy of cancer immunotherapy and overcome resistance to cancer immunotherapy via tumor vessel normalization and intratumoral HEV formation. Shigeta et al. also reported consistent synergism of anti-VEGFR2 and anti-PD-L1 in hepatocellular carcinoma (HCC)^89^. They observed that anti-VEGFR2 therapy upregulates PD-L1 expression under hypoxic conditions, mediated in part by IFN-γ secreted by ECs. Combination therapy with anti-VEGFR2 and anti-PD-1 also promoted durable vascular normalization, which is mediated by PD-1-expressing CD4^+^ cells. Dual combination therapy has also been shown to improve overall survival (OS) and anti-cancer immunity with increased intratumoral accumulation of CTLs and M1-like TAMs. Collectively, combination therapy with anti-VEGFR2 and anti-PD-1 reprograms the immune microenvironment via vessel normalization, further strengthening the anti-cancer immune response and overcoming resistance to cancer immunotherapy in HCC.

Anti-angiogenic therapy can also overcome resistance to anti-PD-1 by abolishing the TOX-mediated T-cell exhaustion program in the TME^24^. Kim et al. revealed that VEGF significantly upregulates the transcription factor TOX, which influences the phenotype and function of CTLs. The TOX-mediated transcriptional program resulted in severe T-cell exhaustion and upregulated inhibitory immune checkpoint receptors such as PD-1, TIM-3, LAG-3, and TIGIT and reduced the proliferation of cytokine production by CTLs. Combination treatment with anti-VEGFR2 and anti-PD-1 enhanced the immunotherapeutic efficacy and T-cell reinvigoration. Collectively, combinatory treatment with anti-angiogenic agents and ICIs is a potential therapeutic option in anti-PD-1-resistant cancer.

Modulating another important angiogenic pathway, ANGPT2/Tie2, has also demonstrated promising preclinical efficacy when combined with anti-VEGF and anti-PD-1. Schmittnaegel et al. demonstrated that combined blockade of VEGF-A and ANGPT2 by a bispecific antibody (A2V) enhanced the therapeutic activity compared with either anti-VEGF-A or anti-ANGPT2 monotherapy alone in both genetically engineered and transplant tumor models^90^. A2V effectively inhibited tumor angiogenesis but promoted vascular maturation in the TME. Moreover, A2V increased tumor antigen presentation by DCs and activated tumor antigen-specific CD8^+^ CTLs, remodeling intratumoral immunity. Although A2V enhanced perivascular CD8^+^ CTL accumulation, it also upregulated PD-L1 expression on tumor vessels via IFN-γ-mediated negative feedback. This negative feedback mechanism was successfully overcome by combining A2V with anti-PD-1, leading to better immunotherapeutic efficacy. These results encourage further testing of combining ICIs with various anti-angiogenic targets other than VEGF in advanced cancers.

Recently, a novel immunotherapeutic target, simulator of IFN genes (STING), was reported to be involved in the regulation of the tumor vasculature and demonstrated synergism with anti-VEGFR2 and ICIs^75^. Yang et al. revealed that intratumoral STING signaling activation suppresses tumor angiogenesis and induces vessel normalization through type I IFN signaling activation and the upregulation of genes related to vascular normalization and endothelial-lymphocyte interaction. Intriguingly, CD8^+^ CTLs are involved in STING-induced vascular remodeling. STING agonist combined with anti-VEGFR2 synergistically enhanced vascular normalization, leading to durable anti-cancer immunity. Notably, STING-based immunotherapy was most effective when combined with anti-VEGFR2 and/or ICIs (either anti-PD-1 or anti-CTLA-4), leading to the complete regression of tumors that are resistant to either anti-angiogenic monotherapy or ICI monotherapy. Therefore, these data suggest that combining novel therapeutics with the combination of anti-angiogenic agents and ICIs could help overcome resistance to anti-angiogenic and immunotherapy in refractory cancers.

On the other hand, immune checkpoint blockade, such as anti-CTLA-4 or anti-PD-1, increases vascular perfusion to improve therapeutic efficacy. Zheng et al. demonstrated that ICI therapy elicits IFN-γ production in CD8^+^ T cells, leading to increased vessel perfusion (IVP)^91^. Notably, IVP can distinguish tumors that are sensitive to ICIs from those that are resistant. In addition, IVP was time-dependently induced by anti-CTLA-4 even before tumor regression was detectable. Collectively, these findings indicate that IVP could be a prerequisite of ICI to improve anti-cancer immunity, thereby enabling it to be used as a predictive indicator for ICI efficacy.

## Clinical evidence for combining anti-angiogenic agents and ICIs in cancer treatment

Preclinical studies continue to yield encouraging results regarding the synergistic effects of ICIs and anti-angiogenic agent combination therapy, which have led to clinical investigations to reproduce these results in patients with advanced cancer^92–98^. Several pivotal clinical trials have already demonstrated the superiority of combining anti-angiogenic agents and ICIs in various malignancies. The most successful results of combination therapy have been reported in renal cell carcinoma (RCC) and hepatocellular carcinoma (HCC).

RCC is a highly immunogenic tumor that has been treated with high-dose IL-2 in some patients. However, its clinical benefit is limited due to the strong toxicity and the limited number of patients who benefit from it, although approximately 10% of patients do achieve a durable response. Immunotherapy has recently been revisited and reevaluated when phase 3 clinical trials demonstrated that nivolumab (anti-PD-1) treatment leads to longer OS with significantly lower toxicity. Research is currently being conducted to maximize the efficacy of immunotherapy by combining PD-1/PD-L1 inhibitors with VEGFR inhibitors. In KEYNOTE-426, patients with previously untreated metastatic RCC were treated with either pembrolizumab (anti-PD-1) and axitinib (VEGFR1, 2, and 3 inhibitor) combination therapy or sunitinib monotherapy, and significantly increased progression-free survival (PFS) was demonstrated in the combination group compared with the sunitinib group^93^. The combination group had a 47% reduced risk of death, and the objective response rate (ORR) of the combination group was 59.3% compared with 35.7% in the sunitinib group. Although the incidence of hepatic toxicity was higher in the combination group, no relevant death event occurred. Based on the significant efficacy and acceptable toxicity profile, combination therapy with pembrolizumab and axitinib was approved by the FDA for treatment-naïve patients with metastatic RCC. JAVELIN Renal 101 (NCT02684006) is a phase 3 clinical trial that evaluated the efficacy of avelumab (anti-PD-L1) and axitinib combination therapy against sunitinib monotherapy in patients with metastatic RCC in a first-line setting^94^. Although the data are premature for OS analysis and require further follow-up, the median PFS of the combination group has already been reported to be 13.8 months compared with 8.4 months for sunitinib. In addition, the ORR and complete response rate were 51.4% vs. 25.7% and 3.4% vs. 1.8%, respectively, showing that these indices have almost doubled. Grade ≥3 toxicity was comparable (71.2% vs. 71.5%) between the two groups. Based on this study, the FDA approved avelumab for use in combination with axitinib as first-line treatment for patients with advanced RCC. Additional clinical trials are ongoing in patients with advanced RCC based on preexisting studies that showed promising results with PD-1/PD-L1 inhibitor and anti-angiogenic agent combination therapy.

In HCC, two highly anticipated phase III studies testing PD-1 inhibitor monotherapy failed to meet their primary endpoints, leading to doubts regarding the use of ICIs in this cancer. However, a randomized phase III clinical trial, IMBRAVE 150 (NCT03434379), demonstrated significant improvements in co-primary end points, PFS and OS, using the combination of atezolizumab (anti-PD-L1) and bevacizumab (anti-VEGF-A) compared with sorafenib^95^. This was the first study to propose a new first-line treatment option that is superior to sorafenib, which has been the standard of care for a decade. This study was initiated from a phase Ib study exploring the efficacy of combining atezolizumab and bevacizumab in patients with various gastrointestinal cancers, including HCC, gastric cancer, pancreatic cancer, and esophageal cancer (GO30140/NCT02715531). At the 2018 ASCO annual meeting, the researchers of this phase Ib study presented that the early-stage ORR was >60% in advanced HCC (investigator-assessed response, 61%; independent review facility-assessed response, 65%)^96^. The FDA granted the Breakthrough Therapy designation based on these data, and the phase III IMBRAVE 150 trial was initiated. At the ESMO Asia 2019 Congress, the median OS with the atezolizumab and bevacizumab combination was not reached until analysis when compared with 13.2 months with sorafenib (*p* = 0.0006); the median PFS was 6.8 months versus 4.5 months (*p* < 0.0001), and the ORR was 27% versus 12% (*p* < 0.0001), respectively^95^. In particular, the ORR of this combination was a huge improvement given that the ORRs for anti-PD-1 inhibitor monotherapy were only 15–20% in patients with advanced HCC. In addition, grade 3–4 adverse events (AEs) were reported in 57% of patients in the combination group compared with 55% of patients in the sorafenib group. In terms of patient-reported outcomes, the combination group exhibited delayed deterioration of quality of life compared with sorafenib. The safety and efficacy of the combination of pembrolizumab and lenvatinib were evaluated in patients with unresectable HCC in KEYNOTE-524, a multicenter, open-label, single-arm phase Ib study^97^. This clinical trial also yielded a promising response rate during the early stage and was granted Breakthrough Therapy designation by the FDA, initiating LEPP-002, a phase 3 trial to evaluate pembrolizumab in combination with lenvatinib as a potential first-line treatment for patients with advanced HCC^97^.

In non-squamous non-small cell lung cancer (NSCLC), a phase 3 clinical trial (Impower150, NCT02366143) comparing atezolizumab (anti-PD-L1), bevacizumab (anti-VEGF), carboplatin, and paclitaxel combination therapy (ABCP group) against bevacizumab, carboplatin, and paclitaxel combination therapy (BCP group) showed significantly extended PFS and OS in the ABCP group compared with the BCP group (median PFS: 8.3 vs. 6.8 months; median OS: 19.2 vs. 14.7 months)^92^. The ORR was significantly higher in the ABCP group than in the BCP group (ORR: 63.5% vs. 48.0%), whereas the adverse event rate was comparable. Based on these results, atezolizumab was approved by the FDA for use in combination with bevacizumab, paclitaxel, and carboplatin as first-line treatment for patients with metastatic non-squamous NSCLC.

Recently, the FDA granted accelerated approval for the use of a combination of pembrolizumab and lenvatinib in patients with advanced endometrial cancer who have experienced disease progression after systemic therapy. This approval was based on the results of the single-arm, multicenter, open-label, multicohort phase Ib/II KEYNOTE-146 trial (NCT02501096)^98^. In this trial, 108 patients who had previously been treated for metastatic endometrial cancer were evaluated for their response to lenvatinib and pembrolizumab. Interim analysis showed that the ORR was 39.6% and 45.3% at 24 weeks by investigator review and independent review, respectively. The most common treatment-related adverse events (TRAEs) of any grade to be reported were hypertension (58%), fatigue (55%), diarrhea (51%), and hypothyroidism (47%). Of the grade 3 TRAEs, the most common were hypertension (34%) and diarrhea (8%), whereas no cases of grade 4 TRAEs were reported. However, immune-mediated AEs, including endocrine, gastrointestinal, hepatic, skin, pulmonary, and renal events, occurred in 55.6% of patients, and 10% of the patients required high-dose glucocorticoids.

In addition to the abovementioned clinical trials, numerous studies are ongoing in various malignancies to prove the efficacy of combining PD-1/PD-L1 inhibitors and anti-VEGF agents (Table 1, https://clinicaltrials.gov). In several years, these ongoing trials are expected to generate consistent results, which will evolve the therapeutic landscape of advanced cancers.

**Table 1:** Ongoing clinical trials investigating combined anti-angiogenic therapy and immune checkpoint inhibitors.

Tumor type	Name (trial ID)	Phase	Setting	*N*	Treatment arm
RCC	CLEAR (NCT02811861)	III	1st	1069	Pembrolizumab + lenvatinib vs. lenvatinib + everolimus vs. sunitinib
RCC	CheckMate9ER (NCT03141177)	III	1st	701	Nivolumab + cabozantinib vs. sunitinib
RCC	CONTACT-03 (NCT04338269)	III	≥2nd	500	Atezolizumab + cabozantinib vs. cabozantinib
HCC	LEAP-002 (NCT03713593)	III	1st	750	Pembrolizumab + lenvatinib vs. lenvatinib
HCC	IMbrave050 (NCT04102098)	III	adjuvant	662	Atezolizumab + bevacizumab vs. active surveillance
HCC	COSMIC-312 (NCT03755791)	III	1st	740	Atezolizumab + cabozantinib vs. sorafenib vs. cabozantinib
NSCLC	NCT02039674	I/II	1st	267	Pembrolizumab + chemotherapy + bevacizumab/ipilimumab/anti-EGFR
Colorectal	COMMIT (NCT02997228)	III	1st	347	Atezolizumab + bevacizumab + mFOLFOX6 vs. bevaziaumb+mFOLFOX6 vs. atezolizumab
Gastric	SEQUEL (NCT04069273)	II	≥2nd	58	Pembrolizumab + ramucirumab + paclitaxel
Melanoma	NCT01950390	II	1st, 2nd	168	Ipilimumab + bevacizumab
Urothelial	NCT03272217	II	1st	70	Atezolizumab + bevacizumab
Solid tumors	NCT02443324	II	≥2nd	155	Pembrolizumab + ramucirumab

*RCC* renal cell carcinoma, *HCC* hepatocellular carcinoma, *NSCLC* non-small cell lung cancer.

## Conclusion

Years of research have demonstrated the potential of ICI monotherapy as well as its limitations, which have led to further attempts to overcome these limitations by combination immunotherapy. Of the potential candidates, the combination of ICI and anti-angiogenic agents continues to yield promising results in both preclinical and clinical studies, not only highlighting that it is one of the most effective combination immunotherapy regimens thus far but also changing the treatment landscape for RCC and HCC. Nonetheless, several issues remain to optimize the efficacy of this combination therapy. First, predictive biomarkers must be developed to identify the subset of patients who will benefit from this combination treatment. Second, the focus on anti-VEGF/R agents as the main anti-angiogenesis agent should be diversified to agents targeting other candidates, such as FGF/R, PDGF/R, and ANGPT2, among others. Third, whether the effects of this combination are synergistic or merely additive must be evaluated. Finally, the angiogenic phenotype differs according to organ; thus, more in-depth analyses must be performed to further our knowledge of the response to ICI treatment at the organ level.
